# Effect of housing system and feed restriction on meat quality of medium-growing chickens

**DOI:** 10.1016/j.psj.2021.101223

**Published:** 2021-04-27

**Authors:** M. Englmaierová, M. Skřivan, T. Taubner, V. Skřivanová, L. Čermák

**Affiliations:** Department of Nutrition Physiology and Animal Product Quality, Institute of Animal Science, Czech Republic

**Keywords:** diet level, pasture herbage, carotenoids, oxidative stability, n3 fatty acids

## Abstract

The aim of the study was to evaluate the differences in meat quality of 420 Hubbard JA757 cockerels in relation to the housing system (litter and mobile box) and level of mixed feed (ad libitum [**AL**], reducing the level by 20% [**R20**] and 30% [**R30**]). Three groups of chickens were housed in litter boxes for the entire fattening period (stocking density: 0.094 m^2^/bird). The other 3 groups were housed in litter boxes until 28 d of age and then relocated into mobile boxes (stocking density: 0.154 m^2^/bird) on pasture until the end of the experiment at 57 d of age. Restricted groups received a reduced diet level from 29th to 57th d of age. Feed mixture restriction increased the pasture vegetation intake of chickens from 2.63 to 3.50 (R20) and 3.94 g of dry matter/bird/d (R30). Restriction adversely affected the dressing percentage (*P* < 0.001) and breast yield (*P* < 0.001), while the leg yield (*P* < 0.001) was increased with increasing restriction levels. Meat of chickens housed in mobile boxes on a pasture showed lower cooking loss (*P* < 0.001) and higher redness and yellowness values in the skin (*P* = 0.030 and *P* = 0.026; respectively) and meat (*P* = 0.008 and *P* < 0.001; respectively). The fragile meat after cooking was observed in chickens reared on litter (*P* = 0.001). As the level of restriction increased, the number of muscle fibres (*P* = 0.001) increased, and their cross-sectional area (*P* = 0.001) and diameter (*P* = 0.002) decreased. The highest contents of lutein (*P* = 0.002) and zeaxanthin (*P* = 0.006) in breast muscle were found in chickens housed in mobile boxes and fed 80% and 70% AL. However, the concentrations of α- and γ-tocopherol (*P* = 0.006 and *P* = 0.003) were negatively affected by feed restriction. A 30% reduction in feed level in outdoor housed chickens led to a decrease in oxidative stability (*P* = 0.024). Feed restriction (R20) in chickens housed in mobile boxes significantly increased the n3 fatty acids content (*P* = 0.002) and h/H index (*P* = 0.005) and reduced the n6/n3 ratio (*P* < 0.001) and atherogenic (*P* < 0.001) and thrombogenic index (*P* = 0.003), which possess a health benefits for human. In addition, restriction of mixed feed decreased cholesterol content in breast meat (*P* = 0.042). It might be concluded that, in terms of meat quality, cereal diet restriction of 20% in medium-growing cockerels housed in mobile boxes on a pasture is beneficial. The higher level of restriction does not lead to further improvement in meat quality indicators.

## INTRODUCTION

Currently, increasing consumer attention is being directed to product quality. The demand for poultry meat with lower growth intensity is increasing. These chickens are mostly used for extensive fattening, which may include free-range with pasture vegetation. Access to pasture may provide fresh grass, clovers and herbs as well as insects that are valued in terms of nutrients (protein and fat) and substances such as chitin or lauric acid that stimulate natural immunity (Glatz et al., 2005; Świątkiewicz et al., 2015; Spranghers et al., 2018; Schiavone et al., 2018; Imathiu, 2020; Kim et al., 2020). Consumption of pasture herbage and plant species composition can favourably influence the meat quality of chickens housed in free-range systems by increasing the content of antioxidants (vitamins and carotenoids) and minerals (Sossidou et al., 2015; Dal Bosco et al., 2016). Furthermore, the level of n3 polyunsaturated fatty acids in meat can be increased in this way (Dal Bosco et al., 2016; Michalczuk et al., 2017) with potential benefits for human health but also stronger tendencies to possible oxidation and rancidity (Michiels et al., 2014). Moreover, vitamin E and other antioxidants present in pasture herbage increase the oxidative stability of meat and thus extend its shelf life while maintaining its sensory properties. Outdoor systems with the possibility of grazing can contribute to the welfare of chickens and also save feed costs.

The pasture herbage intake of chickens depends on the chicken genotype and the composition and dosage of the mixed feed. Slow-growing chickens are more active than fast-growing chickens and show high physical activity and hence the ability to graze. Lorenz and Grashorn (2012) estimated that approximately 10% to 15% of total feed intake may come from pasture in chickens.

The results of Ponte et al. (2008a) suggested that pasture intake (<5% dry matter [**DM**]) had a low impact on the fatty acid and vitamin E homologue profiles of meat from free-range broilers. Restriction of a cereal-based diet led to significantly higher (*P* < 0.05) grazing consumption. The estimated consumption was 1.6%, 2.8%, and 4.9% (on a DM basis) of total feed intake for ad libitum (**AL**)-fed chickens and restricted chickens at levels of 75% or 50% (Ponte et al., 2008b). In addition, pasture intake promoted the consumption of cereal-based feed available for AL consumption by free-range chickens, leading to increase final body weight of chickens of a slow-growing genotype (Ponte et al., 2008c). This is another reason for choosing restrictions in chickens with the possibility of grazing.

On the basis of the findings above, it can be expected that feed restriction in medium-growing chickens will lead to an increase in pasture herbage consumption, which will be reflected in enhancing meat quality, particularly regarding the content of vitamins, carotenoids and n3 fatty acids. The question remains as to what level of restriction will be most appropriate with regard to the chicken genotype, feed, free-range type and resulting effect on meat quality. Therefore, the aim of the present study was to determine differences in the meat quality of medium-growing Hubbard JA 757 chickens in relation to the housing system and dosing of cereal diet. Meat quality was evaluated on the basis of the determination of the physical and muscle fibre characteristics, vitamin and carotenoid contents, fatty acid composition and oxidative stability in meat.

## MATERIALS AND METHODS

### Chickens, Husbandry, and Restrictions

The experiment was performed with 420 one-d Hubbard JA757 cockerels. The cockerels were divided into 6 groups of 70 birds according to the housing system (litter and mobile box) and the feed mixture level (AL, with level reductions of 20% [**R20**] and 30% [**R30**]). The chickens of the 3 groups were kept in indoor pens for the entire fattening period (stocking density: 0.094 m^2^/bird) on wood shavings with ventilation provided by a temperature-controlled fan, gas heating and a 16 h lighting programme. The temperature in the room at arrival was 32°C and continuously decreased to 20°C. Each pen was equipped with pan-feeders and nipple drinkers. The environmental conditions were kept in accordance with the requirements for Hubbard JA757 cockerels. The other 3 groups were housed in indoor pens until 28 d of age and then transported on pasture and housed in floorless portable pens (stocking density: 0.154 m^2^/bird) with dimensions 3.0 × 3.6 × 0.6 m (a total area of 10.8 m^2^ per pen). The pens contained feeders and hat drinkers. The portable pens were constantly moved twice daily to restrict grassland damage, once during the morning feeding at 8:00 h and again at 18:00 h. Throughout the experiment, the chickens were fed 3 mixed feeds. A starter was fed until 28 d of age AL. A grower was fed between 29 and 42 d of age, and a finisher was fed from 43 to 57 d of age. The mixed grower and finisher feeds were fed AL or at a level reduced by 20% or 30% according to treatment. The amount of mixed feed for the restricted groups was calculated from the feed consumption of the ALgroups and separately for indoor and outdoor housing. The ingredients and nutrient contents of the cereal diets and freeze-dried pasture herbage are shown in Tables 1 and 2. Water was provided AL. The grazing part of the experiment was carried out in October 2018, and the average temperature during the monitored period was 11°C. The dominant species of the pasture herbage were *Lolium perenne, Festuca pratensis*, and *Trifolium pratense*. Pasture intake was measured on the 50th d and indirectly assessed by the modified method of Dal Bosco et al. (2014). Pasture herbage samples were collected in square areas (50 × 50 cm) and then calculated for the whole area of each portable pen.

**Table 1:** Diet composition.^1^

Ingredient (g/kg)	Starter	Grower	Finisher
Soybean meal	360.0	272.5	250.0
Maize	140.0	130.0	169.5
Wheat	462.0	560.0	550.0
Sodium chloride	3.0	3.0	3.0
Monocalcium phosphate	13.0	11.0	10.0
Limestone	17.0	18.5	12.5
Vitamino-mineral premix^2^	5.0	5.0	5.0

1 A starter was fed until 28 d of age ad libitum. A grower was fed between 29 and 42 d of age, and a finisher was fed from 43 to 57 d of age. The mixed feeds grower and finisher were fed ad libitum or at a reduced level by 20% or 30% according to treatment.

2 Vitamin-mineral premix provided per kg of diet: vitamin A 10,465 IU, vitamin D3 520 IU, vitamin E 30 IU, menadione 3 mg, thiamine 3 mg, riboflavin 5 mg, pyridoxine 4 mg, cyanocobalamin 40 µg, niacin 25 mg, calcium pantothenate 12 mg, biotin 0.15 mg, folic acid 1.5 mg, choline chloride 250 mg, copper 12 mg, iron 50 mg, iodine 1 mg, manganese 80 mg, zinc 60 mg, selenium 0.3 mg.

**Table 2:** Nutrient content of the diets and freeze-dried pasture.

Analysed nutrient content	Starter	Grower	Finisher	Freeze-dried pasture herbage
AME (by calculation MJ/kg)	12.3	12.1	12.0	5.6
Dry matter (g/kg)	892	889	890	967
Crude protein (g/kg)	206	178	171	162
Fat (g/kg)	16.1	16.3	17.2	33.4
n6/n3	7.4	7.2	7.1	0.36
α-Tocopherol (mg/kg)	35.7	26.6	17.8	97.1
γ-Tocopherol (mg/kg)	6.7	5.4	5.7	8.9
Retinol (mg/kg)	1.84	1.59	0.94	-
Lutein (mg/kg)	0.68	0.66	0.71	73.1
Zeaxanthin (mg/kg)	0.45	0.43	0.44	53.5

Abbreviation: AME, apparent metabolizable energy.

The study was conducted according to the guidelines of the Ethics Committee of the Central Commission for Animal Welfare at the Ministry of Agriculture of the Czech Republic (Prague, Czech Republic) and carried out in accordance with Directive 2010/63/EU for animal experiments. The protocol of this experiment was approved by the Ethical Committee of the Institute of Animal Science (Prague-Uhříněves, Czech Republic, protocol code 04/2018).

At the end of the experiment, at 57 d of age, all chickens were weighed and 8 chickens with an average body weight (average ± 50 g) were selected from each group and slaughtered. After slaughter, bleeding and plucking of the chickens, the feet and head were cut off, and the viscera were removed. The carcasses were stored for 24 h in a refrigerator at 4°C. After cooling, the carcass weight was determined, and carcass analysis was carried out. The breast muscles were separated on the chest from the shoulder joint and sternum. The legs were separated from the torso in the hip joint. The percent composition of these components was calculated as a proportion of the carcass weight. In addition, abdominal fat weight was monitored. The dressing percentage was calculated by dividing the carcass weight by body weight. The breast muscles (*pectoralis major*) were dissected for analyses of meat quality.

### Analyses

The ultimate pH value of breast muscle was detected 24 h *postmortem* using a 330i pH meter (WTW, Weilheim, Germany) with a glass probe introduced 1 cm deep into the transverse section of the breast muscle. The cooking loss of the breast muscle was determined for samples weighing approximately 150 g after 60 min of cooking at 75°C and was calculated from the differences between the weights of the raw and cooked samples. The skin colour and meat colour were measured on a transverse section of the breast muscle 24 h *postmortem* using the Minolta SpectraMagicTM NX analyser (Konica Minolta Sensing, Inc., Osaka, Japan) and are expressed as L*, a* and b*. Meat tenderness (the Warner-Bratzler shear test) in the boiled breast meat was determined by the method showed in the article of Englmaierová et al. (2020). To determine the histochemical parameters of the breast muscle, samples were collected immediately after slaughtering. The samples were frozen in 2-methylbutane cooled by liquid nitrogen (-156°C) and stored at -80°C until histochemical analysis. The samples were cut (cross-sections with a thickness of 12 μm) at -20°C using a Leica CM 1850 cryo-stat (Leica Microsystems Nussloch GmbH, Nussloch, Germany). Subsequently, staining with haematoxylin and eosin for the basic histological characteristics of the muscle fibres was performed. Image analysis NIS Elements AR 3.1 (Laboratory Imaging s.r.o., Prague, Czech Republic) was used to detect the number of muscle fibres per 1 mm^2^, diameter and fibre cross-sectional area.

Analyses of the diet, freeze-dried pasture and breast meat including determination of DM, fat and crude protein were performed by standard AOAC (2005) procedures. The contents of carotenoids and vitamins and lipid oxidation were measured by high-performance liquid chromatography. The high-performance liquid chromatography instrument (VP series; Shimadzu, Kyoto, Japan) was equipped with a diode array detector. The modified method mentioned in the study of Froescheis et al. (2000) was used for lutein and zeaxanthin contents determination. The α-tocopherol, γ-tocopherol and retinol contents were analysed in accordance with the European standards EN 12822 (2000) and EN 12823-1 (2000). The lipid peroxidation levels in breast meat that has been stored at 4°C for 0 and 5 d were measured using the modified method of Czauderna et al. (2011). The lipid oxidative stability was expressed in mg of malondialdehyde (**MDA**) per kg of muscle.

For determination of cholesterol in the meat, lipids were saponified, and the unsaponified matter was extracted with diethyl ether in accordance with ISO 3596:2011. Silyl derivatives were prepared using TMCS and HMDS silylation reagents (Sigma-Aldrich, Prague, Czech Republic) and quantified on a gas chromatograph equipped with a SAC-5 capillary column (Supelco, Bellefonte, USA) that was operated isothermally at 285°C. The fatty acids (**FA**) composition of the breast meat was determined after chloroform-methanol extraction of the total lipids (Folch et al., 1957). Alkaline transmethylation of the FAs was performed (Raes et al., 2003). Gas chromatography of the methyl esters was performed using an HP 6890 chromatograph (Agilent Technologies, Inc.) with a programmed 60 m DB-23 capillary column and a flame ionization detector. The fatty acids were identified by their retention times compared with standards. The methodology of Ulbricht and Southgate (1991) was used to calculate the atherogenic index (**AI**) and the thrombogenic index (**TI**). The hypocholesterolemic/hypercholesterolemic index (**h/H**; ratio between hypocholesterolemic and hypercholesterolemic fatty acids) was calculated according to a formula of Santos-Silva et al. (2002).

### Statistical Analyses

The data were analysed using 2-way analysis of variance (**ANOVA**) with the general linear model (**GLM**) procedure in SAS software (2003). The main effects were the housing system (H), the feed mixture level (R) and the interaction between these 2 factors (R × H). The chicken with an average body weight was the experimental unit (n = 8). All differences were considered to be significant at *P* < 0.05. The results in the tables are presented as the mean and standard error of the mean (**SEM**).

## RESULTS

Pasture herbage intake was increased with the level of restriction. The lowest pasture herbage intake included cockerels fed AL (2.63 g of DM/d/bird). Feed restriction by 20 and 30% increased pasture herbage intake to 3.50 and 3.94 g of DM/d/bird, respectively. Table 3 summarizes the carcass characteristics. A significant interaction of the housing system and feed restriction was found in dressing percentage (*P* = 0.020). The cockerels from litter fed AL showed the highest value of dressing percentage (73.8%), while reduced level of diet by 30% in chickens on pasture decreased dressing percentage to 67.4%. All investigated carcass characteristics were negatively affected by restriction (*P* < 0.001) except for leg yield (*P* < 0.001), where the value was increased with increasing restriction level. Outdoor fattening increased breast yield (*P* = 0.008).

**Table 3:** Carcass characteristics, n = 8.

Housing (H)	Litter			Mobile box				Probability		
Restriction (R)	AL	R20	R30	AL	R20	R30	SEM	H	R	H × R
Body weight (d 57; g)	3,006	2,738	2,591	3,177	2,766	2,640	37.3	0.022	<0.001	NS
Carcass weight (g)	2,219	1,977	1,834	2,327	1,915	1,779	32.0	NS	<0.001	NS
Dressing percentage (%)	73.8^a^	72.2^bc^	70.8^c^	73.3^ab^	69.2^d^	67.4^e^	0.39	<0.001	<0.001	0.020
Breast yield (%)	25.1	22.9	23.5	28.2	24.2	24.1	0.38	0.008	<0.001	NS
Leg yield (%)	29.0	29.80	30.7	27.1	29.9	30.2	0.29	NS	<0.001	NS
Abdominal fat (g)	59.4	49.7	29.0	74.0	43.3	25.2	3.33	NS	<0.001	NS

The feed mixture was provided ad libitum (AL) or was restricted by 20% (R20) and 30% (R30).

a-e Means with different superscripts differ significantly. Abbreviations: NS, not significant; SEM, standard error of the mean.

As is evident from Table 4, a significant (*P* < 0.001) interaction of the housing system and feed restriction was determined in the pH of meat measured after 24 h. The highest pH value was found in 20% restricted chickens reared on pasture (5.38), while the lowest pH (5.19) was also determined in pastured chickens but with a higher restriction level (30%). Breast muscle of chickens housed in mobile boxes on pasture showed lower cooking loss (*P* < 0.001) and had higher values of redness (a*; *P* = 0.008) and yellowness (b*; *P* < 0.001). Access to pasture vegetation also significantly increased the redness (a*; *P* = 0.030) and yellowness (b*; *P* = 0.026) of the breast skin. The tender meat after cooking (Warner-Bratzler test; *P* = 0.001) was observed in chickens fattened on litter. Meat tenderness is also influenced by muscle fibre characteristics. In the breast muscle of chickens, only muscle fibre type IIB (white, fast glycolytic fibres) is present. The restriction significantly influenced the number of muscle fibres per 1 mm^2^ (*P* = 0.001), cross-sectional area (*P* = 0.001) and diameter (*P* = 0.002). The number of muscle fibres was increased, and their area and diameter decreased with increasing levels of restriction.

**Table 4:** Physical characteristics and muscle fibre characteristics of breast meat, n = 8.

Housing (H)	Litter			Mobile box				Probability		
Restriction (R)	AL	R20	R30	AL	R20	R30	SEM	H	R	H × R
pH_24_	5.29^bc^	5.30^b^	5.36^ab^	5.29^bc^	5.38^a^	5.19^c^	0.013	NS	0.038	<0.001
Cooking loss (%)	26.0	26.3	26.1	24.8	23.4	21.8	0.38	<0.001	NS	NS
Skin colour										
L*	66.8	70.0	69.8	68.0	71.9	69.4	0.69	NS	NS	NS
a*	-0.26	0.69	-0.34	1.70	1.49	1.00	0.310	0.030	NS	NS
b*	10.1	11.6	8.8	13.4	13.4	12.8	0.66	0.026	NS	NS
Meat colour										
L*	54.1	51.9	53.2	54.1	53.7	55.4	0.48	NS	NS	NS
a*	-2.56	-2.07	-2.55	-2.04	-2.02	-1.67	0.095	0.008	NS	NS
b*	4.87	5.43	4.75	5.98	7.43	8.08	0.265	<0.001	NS	NS
Warner-Bratzler shear force (N)	18.2	16.6	15.2	21.4	20.9	21.3	0.69	0.001	NS	NS
Muscle fibre (type IIB) characteristics										
Number of fibres (per 1 mm^2^)	304	401	446	280	426	411	16.9	NS	0.001	NS
Cross-sectional area (µm^2^)	2,701	2,082	1,802	2,980	1,967	1,906	120.4	NS	0.001	NS
Diameter (µm)	70.7	61.5	59.0	75.0	61.2	60.8	1.65	NS	0.002	NS

The feed mixture was provided ad libitum (AL) or was restricted by 20% (R20) and 30% (R30). L*, lightness; a*, redness; b*, yellowness.

a-c Means with different superscripts differ significantly. NS, not significant; SEM, standard error of the mean.

The contents of selected carotenoids and vitamins and an indicator of the antioxidative properties of fats in breast muscle are shown in Table 5. Statistically significant interactions were recorded for lutein (*P* = 0.002) and zeaxanthin (*P* = 0.006) content. The highest levels of both carotenoids were found in chickens with access to pasture vegetation together with the application of restricted feeding. The possibility of pasture vegetation intake also increased retinol (*P* < 0.001) and α-tocopherol (*P* = 0.017) content and reduced the γ-tocopherol (*P* = 0.003) content in chicken breast muscle. Moreover, access to pasture vegetation decreased the oxidative stability of fresh meat (*P* = 0.002). The lowest oxidative stability of meat stored for 5 d (*P* = 0.024) was shown in restricted (30%) chickens from pasture.

**Table 5:** Carotenoid and vitamin contents and oxidative stability of fat in breast meat (mg/kg), n = 8.

Housing (H)	Litter			Mobile box				Probability		
Restriction (R)	AL	R20	R30	AL	R20	R30	SEM	H	R	H × R
Lutein	0.043^b^	0.032^b^	0.030^b^	0.059^b^	0.100^a^	0.123^a^	0.0064	<0.001	NS	0.002
Zeaxanthin	0.031^b^	0.021^b^	0.021^b^	0.041^b^	0.073^a^	0.084^a^	0.0048	<0.001	NS	0.006
Retinol	0.039	0.044	0.043	0.052	0.049	0.060	0.0016	<0.001	NS	NS
α-Tocopherol	3.81	3.41	3.13	4.46	3.36	3.94	0.111	0.017	0.006	NS
γ-Tocopherol	0.208	0.192	0.175	0.191	0.147	0.162	0.0047	0.003	0.003	NS
MDA, d 0	0.340	0.326	0.344	0.374	0.382	0.406	0.019	0.002	NS	NS
MDA, d 5	0.364^b^	0.372^b^	0.371^b^	0.427^b^	0.422^b^	0.543^a^	0.023	˂0.001	0.017	0.024

The feed mixture was provided ad libitum (AL) or was restricted by 20% (R20) and 30% (R30).

a-b Means with different superscripts differ significantly. Abbreviations: MDA, malondialdehyde; NS, not significant; SEM, standard error of the mean.

Housing in mobile boxes on pasture increased the polyunsaturated n3 fatty acids content (*P* < 0.001; Table 6). Additionally, the ratio of n6/n3 fatty acids and the atherogenic and thrombogenic indexes were lower (*P* < 0.001) in these groups. The atherogenic and thrombogenic indexes reflect the probability of an increase in pathogenic phenomena such as atheromas and thrombus formation, and a lower value is desirable. Conversely, in the h/H index, which is the ratio between hypocholesterolemic and hypercholesterolemic fatty acids and considers the specific effects of fatty acids on cholesterol metabolism, a higher value is desirable. Access to pasture vegetation increased the h/H index (*P* < 0.001). In addition, a feed restriction of 20% in chickens from mobile boxes significantly increased the n3 fatty acids content (*P* = 0.002) and the h/H index (*P* = 0.005) and decreased the n6/n3 ratio (*P* < 0.001) and the atherogenic (*P* < 0.001) and thrombogenic index (*P* = 0.003) compared to other groups. The level of cholesterol in the breast muscle was influenced only by the restriction level (*P* = 0.042) when the cholesterol content was reduced with a restriction of feed.

**Table 6:** Composition (mg/100 g) and indexes of fatty acid, cholesterol (mg/kg) and fat (g/kg DM) contents in breast meat, n = 8.

Housing (H)	Litter			Mobile box				Probability		
Restriction (R)	AL	R20	R30	AL	R20	R30	SEM	H	R	H × R
ALA	2.2^d^	0.8^d^	0.2^d^	13.7^c^	37.3^a^	23.4^b^	2.25	<0.001	0.001	<0.001
EPA	0.182^b^	0.207^b^	0.082^c^	0.236^ab^	0.283^a^	0.293^a^	0.0138	<0.001	NS	0.004
DHA	3.93^b^	3.43^b^	1.40^c^	4.29^b^	5.31^a^	4.76^ab^	0.223	<0.001	0.001	<0.001
SFA	180^b^	103^c^	60^c^	207^ab^	182^b^	245^a^	11.0	<0.001	0.006	<0.001
MUFA	121^bc^	69^c^	34^c^	234^ab^	193^b^	257^a^	14.0	<0.001	0.044	0.008
PUFA	110^bc^	77^c^	41^c^	144^b^	187^a^	190^a^	9.6	<0.001	NS	<0.001
n3	10.6^c^	7.9^c^	3.0^c^	22.1^bc^	56.4^a^	33.7^b^	3.26	<0.001	0.004	0.002
n6	100^b^	69^bc^	38^c^	121^b^	130^ab^	156^a^	6.8	<0.001	NS	<0.001
n6/n3	9.50^b^	8.76^b^	13.30^a^	5.52^c^	2.53^d^	4.76^c^	0.540	<0.001	<0.001	<0.001
AI	0.666^a^	0.470^c^	0.526^b^	0.431^d^	0.352^e^	0.410^d^	0.0149	<0.001	<0.001	<0.001
TI	1.24^b^	1.08^c^	1.32^a^	0.84^d^	0.55^e^	0.78^d^	0.040	<0.001	<0.001	0.003
h/H	1.50^f^	1.93^d^	1.76^e^	2.17^c^	2.72^a^	2.29^b^	0.059	<0.001	<0.001	0.005
Fat	26.3	26.6	20.3	32.5	21.3	24.4	1.36	NS	NS	NS
Cholesterol	367	313	310	352	345	336	6.5	NS	0.042	NS

The feed mixture was provided ad libitum (AL) or was restricted by 20% (R20) and 30% (R30).

a-e Means with different superscripts differ significantly. Abbreviations: ALA, α-linolenic acid; EPA, eicosapentaenoic acid; DHA, docosahexaenoic acid; DM, dry matter; SEM, standard error of the mean; SFA, saturated fatty acids; MUFA, monounsaturated fatty acids; NS, not significant; PUFA, polyunsaturated fatty acids; AI, atherogenic index; TI, thrombogenic index; h/H, hypocholesterolemic/hypercholesterolemic fatty acid ratio.

## DISCUSSION

AL fattening and housing in mobile boxes on the pasture herbage increased the body weight of chickens at the end of the experiment. This finding corresponds with the study of Ponte et al. (2008c), which showed that pasture intake promotes growth by improving the consumption of cereal-based feed, although the levels of forage intake were low. As the level of restriction increased, the body weight of the chickens was decreased. A similar trend was found by Ponte et al. (2008b), who applied 75% and 50% restrictions. The restriction of diet intake positively influenced pasture herbage intake (from 2.6 to 3.5 and 3.9 g DM/d/bird at 20 and 30% restriction, respectively). The pasture herbage intake of chickens depends on the amount and quality of feed. Lorenz and Grashorn (2012) reported pasture vegetation consumption in chickens and hens at the level of 2 – 5 g of DM per day. A similar increasing trend in pasture consumption after the restrictive measure was shown by Ponte et al. (2008b). Restriction of cereal-based feed intake at the level of 25% led to an increase in relative leguminous pasture intake from 1.6 to 2.8% of the total intake (on a DM basis; Ponte et al., 2008b).

Restriction in chickens housed in mobile boxes on pasture decreased the dressing percentage (*P* = 0.020). Ponte et al. (2008b) identically found that an interaction between pasture intake and feed intake restriction (*P* < 0.01) resulted in a greater decrease in carcass yield in birds consuming pasture at 50% restriction feed intake compared with birds without access to pasture that were subjected to feed intake restriction. Outdoor housing in mobile boxes increased breast yield (*P* = 0.008). The higher (*P* < 0.05) proportion of breast muscle in chickens with access to a grass paddock is also evident from a study conducted by Castellini et al. (2002). Lei and Van (1997) also reported that forced locomotor activity increases the proportion of breast muscle in carcass. In the present study, the proportion of legs increased (*P* < 0.001) with increasing restriction level. The increasing proportion of legs in restricted groups might be caused by higher physical activity in search of feed. As expected, the amount of abdominal fat was significantly lower in restricted chickens than in the groups fed AL. This result was probably due to inhibiting hepatic lipogenesis and elevating fatty acid oxidation (Yang et al., 2010) and decreasing in the number of abdominal adipose cells (Zhong et al., 1995). In addition, it might be caused by fat mobilization for energy supply and abdominal fat might be mobilized more easily during a fasting period (Omosebi et al., 2014).

Restriction increased the number (*P* = 0.001) and decreased the area (*P* = 0.001) and diameter (*P* = 0.002) of muscle fibres. The muscle fibre number in chickens is established before hatching. So, any increase in muscle weight post-hatching is accompanied by increased size of individual myofibres, diameter and elongation (Chen et al., 2007). Accordingly, Koomkrong et al. (2016) stated that the increase of live weight and breast percentage is positively correlated with fibre diameter and area. The body weight (*P* < 0.001) and breast percentage (*P* < 0.001) reduction in the restricted groups may explain the increase in the number of fibres per 1 mm^2^ and the decrease in the area and diameter of muscle fibres in these groups. Rehfeldt et al. (2004) consistently stated that quantitative restriction reduced the area of muscle fibres in chickens at slaughter maturity. Additionally, Li et al. (2007) found that restricted chickens had a smaller muscle fibre area at the end of the experiment at 63 d of age compared to the control group. In contrast, 65% restriction between the 8th and 14th d of age of chickens in a study conducted by Chodová and Tůmová (2017) increased the area of muscle fibres (*P* = 0.026) and their diameter (*P* = 0.041). The differences in the results were probably due to the time of restriction used in each experiment. Fattening of chickens in mobile boxes on pasture reduced cooking loss as well as the tenderness of meat and increased the redness and yellowness of the skin (*P* = 0.030 and *P* = 0.026) and meat (*P* = 0.008 and *P* < 0.001). These findings are consistent with the work of Sun et al. (2013), who found that grazing alone demonstrably reduced the values of cooking loss and increased the values of redness (a*) and shear force of the breast muscle. Additionally, Michalczuk et al. (2014) reported higher values of shear force (*P* ≤ 0.01) in grazing chickens. Castellini et al. (2002) explained the deterioration in the tenderness of meat in chickens kept in free range by the greater motor activity of chickens fattened in this way. Higher values of redness and yellowness of breast muscle and skin in chickens from mobile boxes were probably caused by the pasture herbage intake rich in carotenoids, which are stored in adipose tissue.

Pasture herbage is valued mainly due to the higher content of antioxidants, which are stored in grazing poultry in eggs and meat (Castellini et al., 2006; Mugnai et al., 2009; Skřivan and Englmaierová, 2014; Englmaierová et al., 2020). These include vitamin E and carotenoids, which then usually reduce the sensitivity of unsaturated fatty acids to oxidation. The reduction in feed doses led to higher pasture herbage grazing, which was reflected in a higher proportion of carotenoids in the meat and a deterioration in the oxidative stability of the meat. The decrease in the meat oxidative stability of chickens housed on pasture could be caused by the higher proportion of unsaturated fatty acids in this meat due to the intake of pasture vegetation, which is rich in these fatty acids. The lower oxidative stability of the breast meat of grazing chickens (*P* ≤ 0.05) is also evident from the work of Michalczuk et al. (2014). Moreover, access to pasture vegetation increased the α-tocopherol content and reduced the γ-tocopherol content in chicken breast muscle in the present study. Reductions in γ-tocopherol content (*P* < 0.01) in grazing chickens were similarly observed by Ponte et al. (2008b). In their case, however, the content of α-tocopherol was also reduced (*P* < 0.01). In addition, the application of limited feeding reduced the content of both monitored tocopherols in our study. Thus, dietary vitamin E was probably consumed by the animal to reduce polyunsaturated fatty acid oxidation and to reduce stress caused by lower feed doses. Michiels et al. (2014) showed that free-ranging conditions together with severe feed restriction caused oxidative stress in chickens, illustrated by increased levels of lipid peroxidation in plasma and breast muscle. Additionally, these alterations are probably associated with a substantial decrease in α-tocopherol concentrations in the tissues. On the other hand, Ponte et al. (2008b) did not find a significant effect of restriction on the content of α-tocopherol and γ-tocopherol in the breast muscle of grazing and nongrazing chickens.

Pasture vegetation is also a source of healthy n3 polyunsaturated fatty acids (Sun et al., 2012; Dal Bosco et al., 2016; Michalczuk et al., 2017; Zhang et al., 2017). Ponte et al. (2008b), similar to our results, showed that the levels of n3 polyunsaturated fatty acids (α-linolenic, eicosapentaenoic and docosahexaenoic acids) in breast meat were significantly greater in birds consuming leguminous biomass, which suggests important deposition of α-linolenic acid and the conversion of this n3 precursor to its derivatives in these birds. Restriction of mixed feed in chickens housed in mobile boxes forced the chickens to a higher intake of pasture vegetation, which was also reflected in the composition of fatty acids in breast muscle. In birds without access to pasture, feed restriction resulted in lower α-linolenic acid contents in the meat, whereas in birds from pasture, feed intake restriction increased the deposition of this main n3 polyunsaturated fatty acid in breast meat. This result was similar to the study of Ponte et al. (2008b). A positive effect on increasing the n3 fatty acids content was observed with a restriction of 20% in pastured chickens. The 30% restriction was probably too high and reduced the n3 fatty acid content in meat to the level of the AL group in the mobile box. The adverse effect of long-term restriction is evident from the study of Michiels et al. (2014). Restriction in grazing chickens lasting 7 wk resulted in a reduction in the n3 fatty acid content to the value of indoor housed chickens fed AL. Restriction had a positive effect on the cholesterol content in the breast muscle. In contrast, Ponte et al. (2008b) stated that the total cholesterol concentration was increased in meat from birds subjected to the greatest feed restriction (*P* < 0.01).

## CONCLUSION

In terms of increasing pasture herbage consumption by free-range housed chickens, it is beneficial to reduce the level of cereal diet. However, the undesirable effect of the restriction is a decrease in the performance indicators. Therefore, it is important to find a compromise to ensure an increase in antioxidant and n3 fatty acids contents in meat through restriction but with a tolerable reduction in performance. In medium-growing Hubbard JA 757 cockerels, cereal diet restriction of 20% was sufficient to increase lutein and zeaxanthin content and decrease the n6/n3 ratio and the atherogenic and thrombogenic index while reaching a live weight of 2,766 g at 57 d of age.

## ACKNOWLEDGMENTS

This research was funded by the Ministry of Agriculture of the Czech Republic, grant numbers MZE-RO0718 and QK1910387.

## DISCLOSURES

The authors declare no conflicts of interest.
